# Supplementary material for: Highly selective detection of methanol over ethanol by a handheld gas sensor
Source: Nat Commun. 2019 Sep 16;10:4220. doi: 10.1038/s41467-019-12223-4 (PMC6746816; doi:10.1038/s41467-019-12223-4)
Supplement: Supplementary file 1 — Supplementary Information [file 41467_2019_12223_MOESM1_ESM.pdf]

Supplementary Information

Highly Selective Detection of Methanol over Ethanol by a Handheld Gas Sensor

J. van den Broek *et al.*

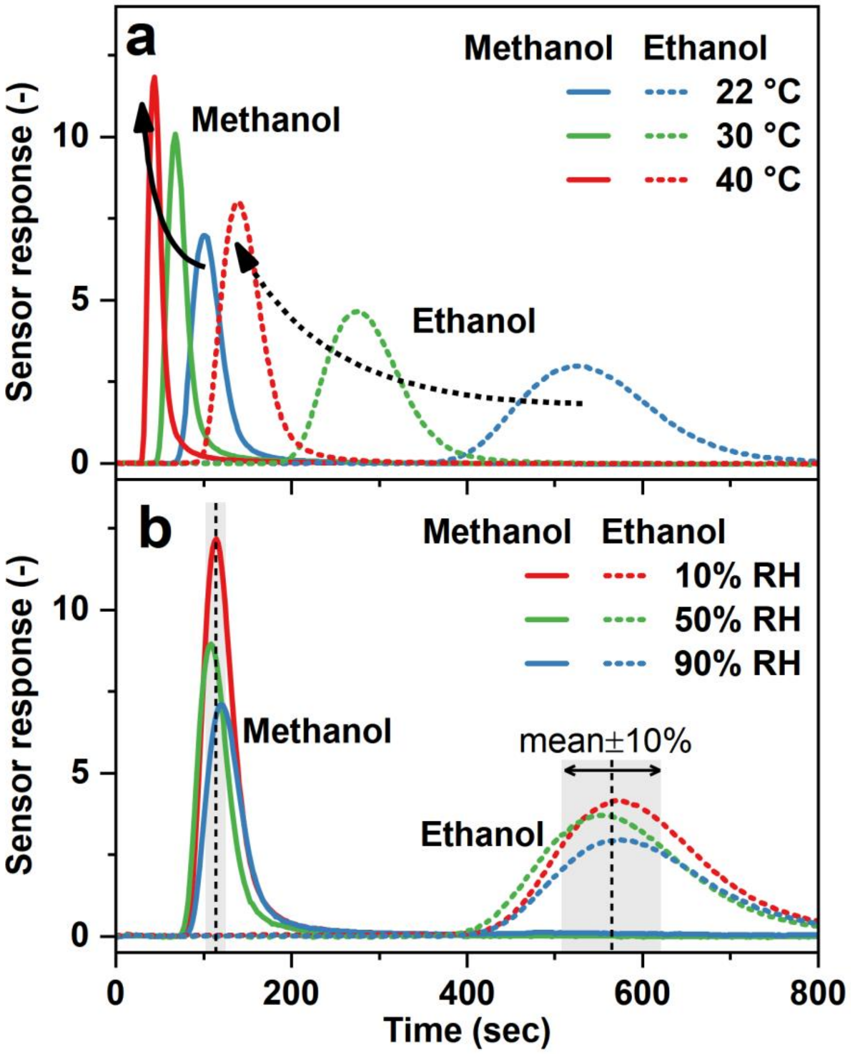

Supplementary Figure 1 | Effect of temperature and relative humidity.

a Response of the methanol detector to 10 sec pulses of 5 ppm methanol (solid lines) and ethanol (dashed lines) at different separation column temperatures of 22 (blue), 30 (green) and 40 °C (red). **b** Response of the methanol detector to 10 sec pulses of 5 ppm methanol (solid lines) and ethanol (dashed lines) at different relative humidity (RH) in the gas flow of 90 (blue), 50 (green) and 10% (red). Dashed black lines and grey shaded areas indicate the mean plus-minus 10% standard deviation of the methanol and ethanol retention times (t_R), respectively.

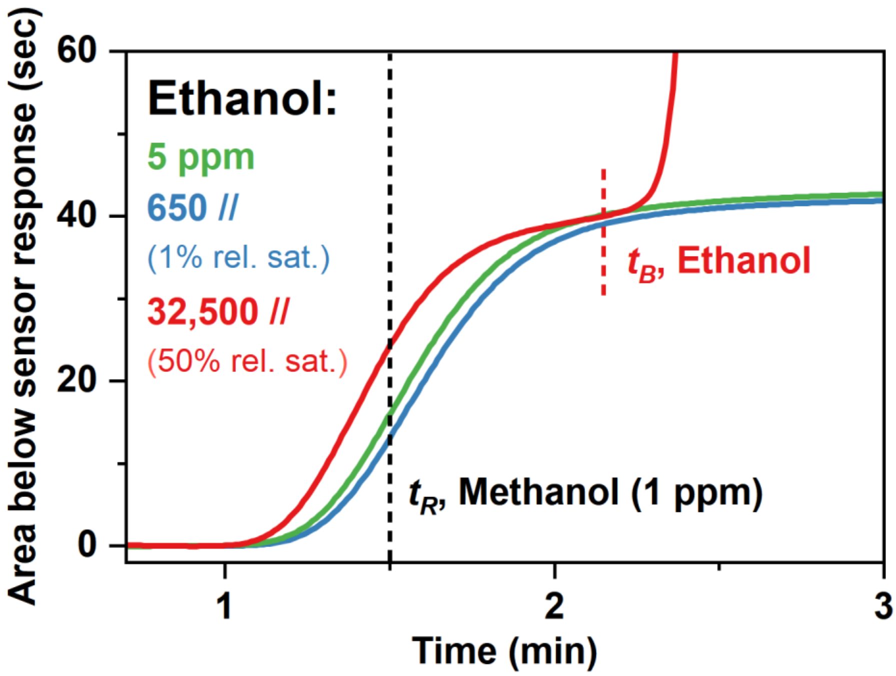

Supplementary Figure 2 | Methanol detection with high ethanol interference.

Area below the response curve of the methanol detector upon exposure to 1 ppm methanol in the presence of 5 (green line), 650 (1% relative saturation, blue line) and 32,500 ppm (50% relative saturation, red line) ethanol. While the maximum peak height is slightly higher with high ethanol interference (see Figure 4a), the areas below the methanol response curves are equal when evaluated just before the breakthrough (t_B, red dashed line) of ethanol. Also shown is the retention time (t_R, black dashed line) of 1 ppm methanol.

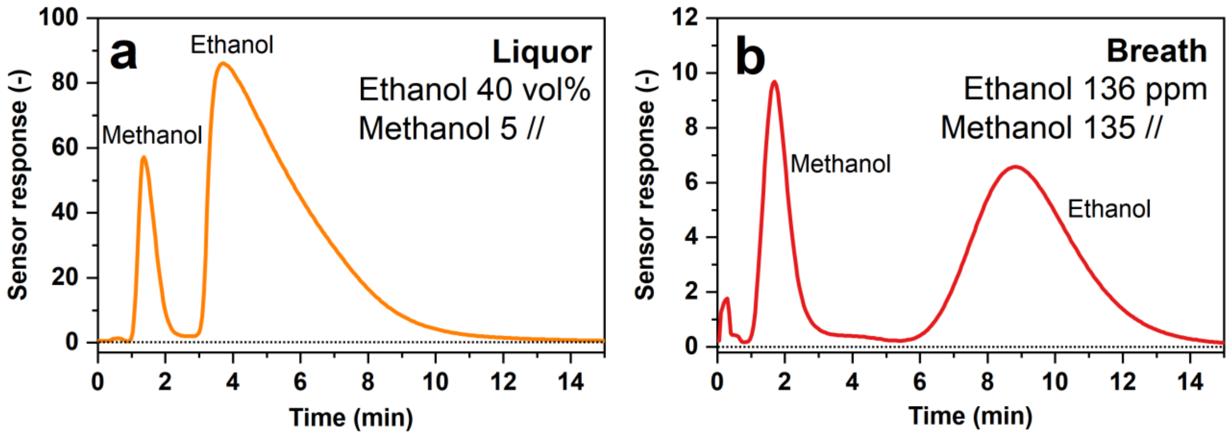

Supplementary Figure 3 | Regeneration after liquor and breath sampling.
Response of the detector to **a** Arrack liquor containing 40 vol% ethanol and laced with 5 vol% methanol (orange line), and **b** breath of an intoxicated volunteer after ingestion of ethanol spiked with 135 ppm methanol (red line) that is equivalent to the breath of a person being intoxicated with the above laced liquor according to the standard addition method[44]. The 136 ppm of ethanol correspond to a blood alcohol level of 0.54‰ according to the Dräger Alcotest 3820 device manual. The sensor baseline (black dotted line) is recovered in both cases within 15 min by flushing with ambient air.

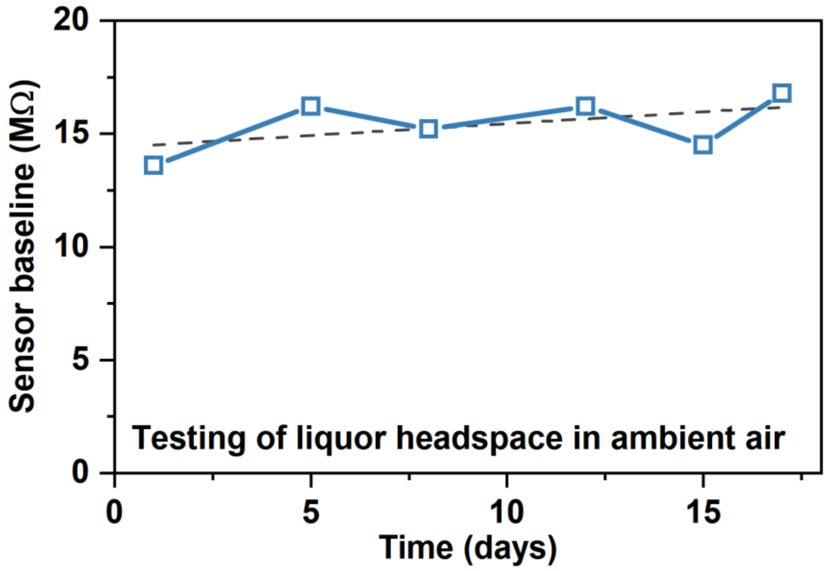

Supplementary Figure 4 | Operational stability in ambient air.
Baseline resistance of the methanol detector in room air over 18 days with regular testing of liquor headspace (blue squares and solid line). A linear fit (black dashed line) indicates an upward drift of 0.7% per day.
